# Translating OMWaNA Trial Findings and Exploring Wider Implications for KMC in Neonatal Care

**DOI:** 10.1002/pdi3.70006

**Published:** 2025-05-08

**Authors:** Aditya Hemendra Bhatt, Somashekhar Marutirao Nimbalkar, Dipen Vasudev Patel, Reshma Kushal Pujara

**Affiliations:** ^1^ Department of Neonatology Pramukhswami Medical College Bhaikaka University Karamsad India

## 1 Introduction

Kangaroo mother care (KMC) represents a groundbreaking intervention in neonatal care that combines skin‐to‐skin contact with exclusive breastfeeding, demonstrating remarkable benefits for infants and mothers. Recent systematic reviews and meta‐analyses have shown that KMC reduces neonatal mortality by 36% among low birth weight infants [1], with an additional 25% reduction when initiated immediately after birth compared to delayed implementation [2]. The intervention significantly decreases the risk of severe infections by 15% and hypothermia by 68% [3] while improving physiological parameters, including oxygen saturation, temperature regulation, and head circumference growth. Studies have demonstrated that preterm infants receiving KMC for more than 90 min daily show lower cortisol levels and greater weight gain [4]. Furthermore, mothers practicing KMC experience a 25% lower risk of moderate to severe depressive symptoms [4], highlighting the dual benefit of this cost‐effective approach. The World Health Organization recommends initiating KMC as soon as possible after birth, particularly for preterm or low birth weight babies, marking a significant global shift in neonatal care practices [1].

Tumukunde et al. [5] recently published a randomized controlled trial (RCT) comparing the efficacy of KMC initiated before clinical stabilization with standard care for neonates in Uganda. This study was necessitated for the high neonatal mortality rates in sub‐Saharan Africa and the potential benefits of KMC documented through previous studies after immediate KMC (iKMC) and early KMC (eKMC) trials [6, 7].

## 2 Study Design and Methodology

The Operationalising kangaroo Mother care before stabilisation amongst low birth Weight Neonates in Africa (OMWaNA) trial, a parallel‐group, individually RCT1 conducted in five government hospitals across Uganda, holds significant implications for neonatal care and public health. The study aimed to compare the effectiveness, safety, costs, and cost‐effectiveness of KMC initiated before clinical stabilization versus standard care in neonates weighing up to 2000 g.

### 2.1 Randomization and Blinding

Randomization was performed using a computer‐generated sequence with permuted blocks of varying sizes, stratified by birthweight and recruitment site. Although parents, caregivers, and healthcare workers were unmasked in treatment allocation, the independent statistician who conducted the analyses was masked. This approach minimized selection bias but introduced potential performance and detection biases due to the lack of blinding among caregivers and healthcare providers [3].

### 2.2 Sample Size and Power

The sample size calculation was based on an anticipated effect size of a 5.6% reduction in 7‐day mortality, assuming a 7‐day mortality rate of 25% in the control group. The study aimed to enroll 2188 neonates to achieve 80% power at a 5% significance level, allowing for 20% attrition. However, the observed mortality rates were lower than expected, which reduced the power to detect the prespecified effect size [5].

### 2.3 Primary and Secondary Outcomes

The primary outcome was all‐cause neonatal mortality at 7 days. Secondary outcomes included all‐cause neonatal mortality at 28 days, hypothermia at 24 h, time to stabilization, time to death, time to exclusive breastmilk feeding, duration of hospital admission, readmission frequency, daily weight gain, and maternal well‐being and responsiveness at 28 days. The choice of primary and secondary outcomes was appropriate, considering the high neonatal mortality rates in sub‐Saharan Africa and the potential benefits of KMC [5].

### 2.4 Statistical Analysis

Using modified Poisson regression models, risk ratios (RRs) with 95% confidence intervals (CIs) were estimated for 7‐day and 28‐day mortality RRs. The complier average causal effect on mortality was also calculated to assess the efficacy of KMC among babies who received KMC within 24 h of stabilization. Subgroup analyses explored the impact of KMC on mortality by gestational age, birth weight, size for gestational age, singleton versus twin birth, hospital site, neonatal mortality risk (NMR) 2000 score, and mode of delivery. The statistical methods were robust and appropriate for the study design [5].

## 3 Results and Interpretation

The trial enrolled 2221 neonates, with 1110 assigned to the intervention group and 1111 to the control group. From randomization to 7 days of age, 81 (7.3%) neonates in the intervention group and 83 (7.5%) in the control group died (adjusted RR 0.97 [95% CI 0.74–1.28] and *p* = 0.85). From randomization to 28 days of age, 119 (10.7%) neonates in the intervention group and 134 (12.1%) in the control group died (RR 0.88 [95% CI 0.71–1.09] and *p* = 0.23). The primary outcome showed no significant difference between the groups, indicating that KMC initiated before stabilization did not significantly reduce early neonatal mortality [5].

However, secondary outcomes, such as hypothermia at 24 h and daily weight gain at 28 days, showed significant improvements in the intervention group. The proportion of neonates with hypothermia at 24 h was 40.9% in the intervention group and 53.1% in the control group (adjusted RR 0.76 [95% CI 0.70–0.83] and *p* < 0.0001). Mean weight gain at 28 days was 7.8 g per day in the intervention group and 7.1 g per day in the control group (adjusted mean difference 0.75 g/day [0.01–1.49] and *p* = 0.047) [5].

### 3.1 Economic Evaluation

The economic evaluation indicated that KMC before stabilization was cost‐effective compared to standard care from societal and provider perspectives. The intervention was expected to be cost saving to providers (adjusted mean difference US$ −57.0 [95% CI −69.9 to −44.1] and *p* < 0.0001) and society (US$ −66.2 [95% CI −85.7 to −46.7] and *p* < 0.0001) in settings where KMC before stabilization significantly reduced the length of hospital stay from 7.3 to 6.1 days [5].

In India, community‐initiated KMC (ciKMC) [8] has been shown to lower healthcare out‐of‐pocket expenditures and reduce families' risk of impoverishment, demonstrating its financial benefits alongside health improvements. For example, ciKMC reduced early infant mortality by 25% (hazard ratio, HR 0.75) while lowering healthcare costs by $5.5 per infant during an 8‐week observation period.

Policy implementation in resource‐limited settings faces challenges such as inadequate infrastructure, cultural resistance, and lack of trained staff. However, evidence suggests that integrating KMC into national health systems through dedicated KMC units, strong government leadership, and community engagement can enhance its feasibility. Cost‐effectiveness is further amplified by reducing reliance on expensive technology and prolonged hospital stays. Tailored strategies, such as family education, ambulatory follow‐up, and capacity‐building for healthcare workers, can optimize KMC in diverse contexts. Scaling up KMC requires political commitment, dedicated funding, and infrastructure improvements. Its affordability and adaptability make it a critical intervention for improving neonatal survival in low‐resource environments.

### 3.2 Comparing iKMC, OMWaNA, and eKMC Trials

The iKMC trial [6] was a RCT conducted in five tertiary‐level hospitals in India, Ghana, Malawi, Tanzania, and Nigeria. It included 3211 neonates with birth weights between 1000 and 1800 g. Participants were randomized to receive either immediate continuous KMC (intervention) or care in an incubator or radiant warmer until stabilization as conventional care, followed by KMC (control). Neonatal mortality at 28 days and 72 h were the primary outcomes. The trial found that neonatal mortality at 28 days was significantly lower in the intervention group (12.0%) compared to the control group (15.7%), with a relative risk of RR, 0.75 (95% CI, 0.64–0.89 and *p* = 0.001). However, the difference in mortality at 72 h was not statistically significant (4.6% vs. 5.8%; RR, 0.77; 95% CI, 0.58–1.04; and *p* = 0.09). Secondary outcomes showed lower incidences of hypothermia (RR, 0.65 and 95% CI, 0.51–0.83) and suspected sepsis (RR, 0.82 and 95% CI, 0.73–0.93) in the intervention group. This trial did not complete the recruitment of the sample size. It was stopped earlier by the Data Safety Monitoring Board due to the observed benefit in neonatal survival among infants receiving immediate KMC.

The eKMC trial [7] was a RCT conducted in a single hospital in The Gambia. It included 277 neonates with birth weights < 2 kg and clinical instability. Participants were randomized to receive either early KMC before stabilization (intervention) or conventional standard care until stabilization, followed by KMC (control). The primary outcome in this study was neonatal mortality at 28 days. The trial found no significant difference in 28‐day neonatal mortality between the intervention (16.7%) and control groups (19.6%) (RR, 0.84; 95% CI, 0.55–1.29; and *p* = 0.43). Secondary outcomes, such as hypothermia and suspected sepsis, also showed no significant differences between the groups. The trial was underpowered due to improvements in the general quality of care, which contributed to a 50% reduction in neonatal mortality in the control group compared to pretrial NMR. The characteristic features of the above studies are in Table 1, and the primary and key secondary outcomes are in Table 2.

**TABLE 1:** The characteristic feature of the above studies.

Characteristic features	iKMC trial	eKMC trial	OMWaNA trial
Study design	Randomized controlled trial	Randomized controlled trial	Randomized controlled trial
Setting	Five hospitals in Ghana, India, Malawi, Nigeria, and Tanzania (NICU of the tertiary‐level hospital)	One hospital in the Gambia (special care baby unit)	Five hospitals in Uganda (neonatal units)
Dates	From November 2017 to January 2020	From November 2018 to March 2020	From October 2019 to July 2022
Sample size	3211 neonates	277 neonates	2221 neonates
Inclusion criteria	Birth weight 1.0–1.8 kg	Birth weight < 2 kg, unstable	Birth weight 0.7–2.0 kg, < 48 h old, unstable
Intervention	Immediate continuous KMC	Early KMC before stabilization	KMC initiated before the stabilization
Control	Conventional care until stable, then KMC	Standard care until stable, then KMC	Standard care until stable, then KMC

Abbreviations: eKMC, early kangaroo mother care; iKMC, immediate kangaroo mother care; OMWaNA, The Operationalising kangaroo Mother care before stabilisation amongst low birth Weight Neonates in Africa.

**TABLE 2:** The primary and key secondary outcomes of the above studies.

Primary and key secondary outcomes	iKMC trial	eKMC trial	OMWaNA trial
Neonatal mortality at 28 days	12.0% in intervention and 15.7% in control RR, 0.75 (95% CI, 0.64–0.89) and *p* = 0.001	16.7% in intervention and 19.6% in control RR, 0.84 (95% CI, 0.55–1.29) and *p* = 0.43	10.7% in intervention and 12.1% in control RR, 0.88 (95% CI, 0.71–1.09) and *p* = 0.23
Neonatal mortality at 72 h	4.6% in intervention and 5.8% in control RR, 0.77 (95% CI, 0.58–1.04) and *p* = 0.09	—	—
Neonatal mortality at 7 days	—	—	7.3% in the intervention group and 7.5% in both the control group RR, 0.97 (95% CI, 0.74–1.28) and *p* = 0.85
Hypothermia	Lower in intervention RR, 0.65 (95% CI, 0.51–0.83)	No significant difference	Lower in intervention at 24 h RR 0.76 (95% CI, 0.70–0.83) and *p* < 0.0001
Suspected sepsis	Lower in intervention RR, 0.82 (95% CI, 0.73–0.93)	No significant difference at 28 days	—
Exclusive breastfeeding	No significant difference	—	No significant difference at 28 days
Weight gain	No significant difference	—	Higher in intervention at 28 days Mean difference 0.75 g/day (95% CI, 0.01–1.49) and *p* = 0.047
Time to stabilization	No significant difference	—	Longer in intervention HR, 1.31 (95% CI, 1.13–1.53) and *p* = 0.0004

Abbreviations: eKMC, early kangaroo mother care; HR, hazard ratio; iKMC, immediate kangaroo mother care; OMWaNA, The Operationalising kangaroo Mother care before stabilisation amongst low birth Weight Neonates in Africa; RR, risk ratio.

### 3.3 Strengths and Limitations

The study's strengths include a robust design with a large sample size of 2221 neonates and comprehensive data collection, enhancing the reliability and generalizability of its findings (Table 3). It provides valuable insights into the cost‐effectiveness of immediate KMC initiated before clinical stabilization through detailed economic evaluations from societal and provider perspectives. The trial's implementation in real‐world settings across five government hospitals in Uganda strengthens its applicability to similar contexts in sub‐Saharan Africa, with thorough ethical oversight ensuring moral conduct and reporting.

**TABLE 3:** Summary of the OMWaNA trial.

Population/problem	Neonates weighing up to 2000 g, aged younger than 48 h, without life‐threatening clinical instability.
Intervention	Kangaroo mother care was initiated before clinical stabilization.
Comparison	Standard care (incubator or radiant heater care until stability criteria were met).
The frame of outcome measurement	Up to 28 days following hospital admission.
Setting	Five hospitals across Uganda.
Clinical question	In neonates weighing up to 2000 g, what is the effectiveness and cost‐effectiveness of KMC initiated before clinical stabilization compared to standard care on neonatal mortality and other clinical outcomes within 28 days of admission?
Inclusion criteria	Singleton or twins < 48 h, weighing 0.7–2 kg, without any life‐threatening clinical instability.
Exclusion criteria	Triplet or higher multifetal pregnancy, parent or caregiver unable or unwilling to consent/perform KMC or attend follow‐up visits, neonates with life‐threatening instability, severe jaundice requiring immediate management, active seizures, or major congenital malformation.
Recruitment procedure	Not specified.
Randomization	Computer‐generated random allocation sequence with permuted blocks of varying sizes, stratified by birthweight and recruitment site.
Allocation concealment	The allocation sequence programmed into the screening database is revealed only after screening completion.
Blinding	Parents, caregivers, and healthcare workers were unmasked about treatment allocation, and the independent statistician who conducted the analysis was masked.
Execution of intervention	KMC: Nnonates are placed prone and skin‐to‐skin on the caregiver's chest, secured with a KMC wrap. Standard care: neonates were cared for in an incubator or radiant heater until stability criteria were met.
Outcomes	Primary: All‐cause neonatal mortality at 7 days. Secondary: All‐cause neonatal mortality at 28 days, hypothermia at 24 h, time to stabilization, time to death, time to exclusive breastmilk feeding, duration of hospital admission, readmission frequency, daily weight gain at 28 days, women's wellbeing, and maternal responsiveness at 28 days.
Follow‐up protocol	Follow‐up visits or oral reports from the caregiver at 28 days
Sample size	2221 neonates (1110 in the intervention group and 1111 in the control group)
Data analysis	Intention‐to‐treat analysis, modified Poisson regression models, cox proportional hazards regression models, and linear regression models.
Comparison of groups' baseline	Similar age distribution, gestational age, birth weight, sex ratio, multiple births, mode of delivery, birth location, and maternal characteristics between the intervention and control groups.

However, the study has several limitations. The primary outcome of early neonatal mortality was not significantly reduced by KMC compared to standard care, potentially limiting its perceived effectiveness and influence on policy decisions. The lack of blinding for parents, caregivers, and healthcare workers could introduce bias, affecting internal validity. Variability in KMC implementation across different hospitals might reduce the consistency and reliability of findings. The follow‐up period was limited to 28 days, possibly missing long‐term outcomes and benefits. A 20% attrition rate assumption could impact data robustness, leading to potential biases. Results are specific about Ugandan healthcare, limiting generalizability to other regions. The lack of detailed data on some secondary outcomes hinders comprehensive understanding. Unmeasured variables might influence outcomes despite adjustments for confounding factors. The economic evaluation's conclusions depend on assumptions and estimates without modeling costs over a lifetime horizon, affecting the reliability of the cost‐effectiveness assessment.

## 4 Conclusion

This well‐designed RCT showed that KMC initiated before clinical stabilization in neonates resulted in a similar early neonatal mortality rate to those receiving standard care. However, secondary outcomes, such as hypothermia and weight gain, showed significant improvement in the KMC group.

The OMWaNA trial, published in *The Lancet*, assessed the impact of initiating KMC before clinical stabilization on neonatal mortality at 7 and 28 days but found no statistically significant differences. Mortality rates were 7.3% in the intervention group and 7.5% in both the control group at 7 days and slightly lower in the KMC group at 28 days (10.7% vs. 12.1%), with a relative risk of 0.88 (95% CI, 0.71–1.09 and *p* = 0.23). However, lower‐than‐expected baseline mortality rates limited the trial's statistical power, which reduced the ability to detect small but meaningful effects. The study's sample size, though substantial (2221 neonates), was insufficient to identify modest reductions in mortality due to smaller observed effect sizes and fewer events than anticipated. This limitation suggests that clinically relevant benefits of prestabilization KMC may have gone undetected. The findings highlight the importance of accurate baseline assumptions during study design and the need for larger sample sizes or pooled analyses to improve power. Although the trial concluded no significant mortality reduction, trends favoring KMC suggest potential benefits warranting further investigation, particularly in higher‐risk populations or through alternative statistical approaches to interpret nonsignificant results better. The issues highlighted above and the frequent use of combinations of both medications necessitate additional data analysis and external validation before the two care methods can be considered equivalent.

## Author Contributions

**Aditya Hemendra Bhatt:** conceptualization, formal analysis, writing – original draft. **Somashekhar Marutirao Nimbalkar:** conceptualization, formal analysis, writing – review and editing, supervision, visualization, validation. **Dipen Vasudev Patel:** visualization, writing – review and editing. **Reshma Kushal Pujara:** validation, writing – review and editing. All authors approved the final manuscript as submitted and agree to be accountable for all aspects of the work.

## Ethics Statement

The authors have nothing to report.

## Conflicts of Interest

The authors declare no conflicts of interest.

## Data Availability

The data that support the findings of this study are openly available in Lancet/pubMed at https://www.thelancet.com/journals/lancet/article/PIIS0140‐6736(24)00064‐3/fulltext.
